# A comparative analysis of response times shows that multisensory benefits and interactions are not equivalent

**DOI:** 10.1038/s41598-019-39924-6

**Published:** 2019-02-27

**Authors:** Bobby R. Innes, Thomas U. Otto

**Affiliations:** School of Psychology & Neuroscience, St. Mary’s Quad, South Street, St. Andrews, KY16 9JP United Kingdom

## Abstract

Multisensory signals allow faster responses than the unisensory components. While this redundant signals effect (RSE) has been studied widely with diverse signals, no modelling approach explored the RSE systematically across studies. For a comparative analysis, here, we propose three steps: The first quantifies the RSE compared to a simple, parameter-free race model. The second quantifies processing interactions beyond the race mechanism: history effects and so-called violations of Miller’s bound. The third models the RSE on the level of response time distributions using a context-variant race model with two free parameters that account for the interactions. Mimicking the diversity of studies, we tested different audio-visual signals that target the interactions using a 2 × 2 design. We show that the simple race model provides overall a strong prediction of the RSE. Regarding interactions, we found that history effects do not depend on low-level feature repetition. Furthermore, violations of Miller’s bound seem linked to transient signal onsets. Critically, the latter dissociates from the RSE, demonstrating that multisensory interactions and multisensory benefits are not equivalent. Overall, we argue that our approach, as a blueprint, provides both a general framework and the precision needed to understand the RSE when studied across diverse signals and participant groups.

## Introduction

**Figure 1:**
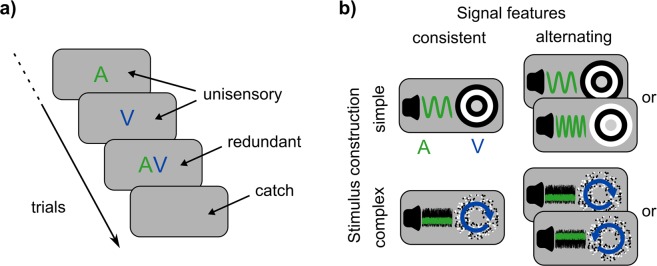
Experimental design. (**a)** Redundant signals paradigm. Participants respond to trials with auditory (A), visual (V), and combined signals (AV), but not on catch trials. Signals were presented in a random order within a block of trials. (**b)** Following a 2 × 2 design, we tested four signal sets in separate blocks. Stimulus construction was either simple (e.g. a pure tone, in audition) or complex (e.g. a noise tone). The sequence of signal features was either consistent (e.g. one frequency) or alternating (e.g. randomly one of two frequencies).

Everyday life requires processing of multiple signals coming from different sensory modalities like audition and vision. An incoming phone call, for example, can be signalled either in one modality only (e.g. a ringtone as a unisensory signal) or in multiple modalities simultaneously (e.g. a ringtone and a flashing screen as redundant signals). As observed in the redundant signal paradigm (Fig. 1a), the benefit of redundant signals is that individuals respond faster compared to the unisensory components, which is the *redundant signals effect* (RSE) as described in classic experiments^1–3^.

Given the broad scope of multisensory benefits, the RSE has been investigated for over 100 years with different research questions and diverse signal sets. Basically all sensory modalities have been tested, including audio-tactile^4^, visual-tactile^5^, and gustatory-olfactory pairings^6^. The largest body of research has focused on audio-visual pairings, but even within this domain, stimuli have varied considerably across studies. Some have used abstract signals, such as pure tones or noise sounds that were paired with flashes, letters, or simple geometric shapes^7–9^. Others have used real-world signals, such as animal sounds and images^10^ or signals with emotional content^11^. In most experiments, signals have had sudden-onsets and were presented briefly with little variation across trials, but the RSE was also observed when signals were randomly generated on each trial and presented within a continuous audio-visual background^12,13^. While the basic RSE has reliably replicated, the diverse studies differ in several aspects including overall mean RTs and the exact size of the RSE. Due to these differences, a recent review argued that a common modelling framework for the RSE is unlikely to be found^14^. In this view, a comparative approach between studies is difficult and a complete understanding of the RSE is lacking.

To develop a common framework, it is a good starting point to build upon models of unisensory decision-making, which typically assume some key processing steps e.g.^15–17^. Sensory evidence for a signal is available from the environment, and is sampled by neurons with corresponding receptive fields. Due to fluctuations in neuronal activity, the evidence is subject to *noise*. To minimise the influence of noise on signal detection, the evidence is accumulated over time until a threshold level is reached and a response is triggered. To extend the framework to the RSE, some sort of *combination rule* is needed that specifies how evidence from two senses is processed to trigger a single response. Broadly, there are two main model architectures with fundamentally different combination rules. Firstly, so-called *race models* suggest that evidence for each signal is accumulated separately by parallel decision units (e.g. one for audition and one for vision) and that the first unit to reach its threshold triggers a response. Secondly, *pooling models* propose that evidence for both signals is summed in a single decision unit and that a response is triggered when the combined evidence reaches a threshold level. Both architectures could in principle explain the RSE.

The race model architecture was first proposed by Raab^18^. The idea is that selecting the faster of two stochastic decision processes results on average in faster and less variable RTs with redundant compared to unisensory signals. Thus, race models explain the RSE mainly by statistical facilitation. Miller^8^, however, argued that if race models were correct, an upper bound for the RSE can be computed on the level of RT distributions, which is named Miller’s bound. As this bound is typically violated in experiments, Miller^8^ and many follow-up studies concluded that race models cannot explain the RSE, which has led to the dominance of pooling models. However, to the best of our knowledge and in agreement with the recent review^14^, no pooling model has been shown to account for the RSE across studies. Hence, the lack of a common framework is in essence a failure of the dominating class of pooling models. The possibility that race models provide a common framework remained unexplored due to the general rejection.

This omission becomes critical as the general rejection of race models is not a valid argument^19^. The issue is the untested and mostly neglected *context invariance* assumption, which states that the processing of one signal is not affected by another^20,21^. As Miller’s bound implicitly assumes context invariance, a violation of the bound does not necessarily show that the basic race model architecture is wrong. Alternatively, the context invariance assumption may be wrong, which would show that some sort of interaction has taken place between the parallel decision units. If the context invariance assumption is dropped, we demonstrated that context variant race models can explain the RSE, including violations of Miller’s bound^12,13^. These considerations show that the parallel architecture of race models could in fact provide a common framework for RSE studies.

To study the RSE with diverse signal sets, here, we develop a comparative approach based on race models. In a first of three steps, we test the ability of the framework to predict multisensory benefits. Following Raab’s model^18^, directional predictions of the RSE for different sets of signals can be readily made according to the *principles of multisensory behaviour*^12^. Firstly, the *principle of equal effectiveness* states that benefits should increase when unisensory RTs become more similar. Secondly, the *variability rule* states that the variability of RTs is the key driving force of multisensory benefits, which should increase when unisensory RTs become more variable. In addition, following Raab’s model^18^, a parameter-free prediction can be made of the exact size of the RSE based on the unisensory RT distributions and *probability summation*. The first step thus provides a simple tool to analyse the RSE if diverse signal sets yield different RTs.

The second step quantifies two processing interactions with multisensory signals that are not covered by Raab’s model. The first interaction concerns *history effects* that describe the influence of previous trials on current-trial processing^4,8,13^. Typically, RTs to unisensory signals are faster if a modality is repeated (e.g. audition following audition) compared to a switch (e.g. audition following vision). This interaction challenges the *statistical independence* assumption that is made by the parameter-free prediction using probability summation^19^. The second interaction is observed by violations of Miller’s bound, which shows that processing of one signal must affect processing of the other signal in some way if the parallel processing architecture is correct. This second interaction challenges the *context invariance* assumption made by the parameter-free prediction based on probability summation^19^. By quantifying the two interactions, it becomes clear that Raab’s basic model needs to be extended. Moreover, the interactions need to be critically considered to understand how multisensory interactions are eventually changed with diverse signals.

The third step is to account for the RSE on the level of entire RT distributions with a modelling approach. Specifically, we use here the context variant race model that includes two free parameters to account for the interactions^12,13,19^. Regarding history effects, the model includes the correlation *Rho*, which is used in the probability summation rule given that the assumption of statistical independence does not hold. Regarding violations of Miller’s bound, the model includes the noise *Eta*, which models increased noise within the accumulation process for redundant compared to unisensory conditions. This increased noise may come, for instance, from a disruptive interaction between parallel decision units (i.e. having both accumulate evidence at the same time decreases their reliability). This second parameter is motivated by findings showing that empirical RT distributions with redundant signals are not faster but more variable than the best-fitting race model with only *Rho* as a free parameter^13^. A key advantage of considering entire RT distributions is that these provide a more detailed view compared to an approach that is based only on a subset of particularly fast RTs as in Miller’s test. Such a detailed view is critically needed given that present understanding of multisensory interactions is still far from complete. For example, the source of additional noise is not yet known and subject to speculation^19^. Thus, it will be particularly important to identify experimental manipulations that specifically target one or the other interaction so that the underlying sources can be determined. In the end, we are convinced that such a comparative approach will lead to a better understanding of multisensory processing.

Following the comparative approach, we developed stimuli to mimic some of the diversity across RSE studies. Using a 2 × 2 design, we manipulated *stimulus construction* and *signal features* in each modality (Fig. 1b). Regarding the first factor, the construction of auditory and visual stimuli was either simple (identical across trials; without background stimulation) or complex (randomly generated; with background stimulation). One aspect of this manipulation is that simple stimuli had strong onset transients, which were masked in complex conditions by the background. As onset transients have been considered relevant to multisensory processing^22,23^, we aim to evaluate whether processing interactions, as revealed by the modelling approach, change according this factor. Regarding the second factor, signal features were either consistent (one variant per modality) or alternating (one of two variants per modality). With this factor, we aimed to understand the origins of history effects. One possibility is that history effects arise simply because of the repetition of low-level features (e.g. the specific frequency of a tone). If true, introducing alternating signals within each modality would reduce history effects. Alternatively, if no changes in history effects occur, this would suggest a basis in higher-level processes.

## Methods

### Participants

20 healthy adults (18–29 years; 14 females) were recruited via the University of St Andrews. All were naïve regarding the experiment’s purposes and reported normal hearing and normal/corrected-to-normal vision. Informed consent was obtained from all participants. Participants were reimbursed with £9. All procedures were approved by the University of St. Andrews’ *University Teaching and Research Ethics Committee* (UTREC, approval code: PS12181) and were performed in accordance with the Code of Human Research Ethics (British Psychological Society, 2014).

### Apparatus

The experiment was controlled by a Dell computer (Optiplex XE2) equipped with Matlab and the Psychophysics Toolbox extensions^24–26^. Visual stimuli were presented on a Dell UltraSharp U2713HM monitor (resolution: 1,920 × 1,080 pixel; refresh: 60 Hz). Viewing distance was 57 cm. Auditory stimuli were presented via over-ear headphones (Sennheiser HD 280 Pro) at a 44.1 kHz sample frequency. The volume was calibrated using a Brüel and Kjær sound level meter (Type 2250) attached to an artificial ear (Type 4153). Participants responded by pressing a custom-built handheld button connected to an RTbox V5^27^. Prior to data collection, the RTbox was also used to calibrate audio-visual timing. As a test, audio-visual signal onsets were presented in 1000 trials. Calibrated stimuli were synchronous and onset jitter was reliably below 1 ms.

### Task

We used the redundant signals paradigm (Fig. 1a). On signal trials (75%), we presented either an auditory (A), a visual (V), or redundant signals (both auditory and visual together, AV). Participants pressed a button as quickly as possible after detecting any signal. On catch trials (25%), no signal was presented and participants were asked to withhold a response.

### Stimuli

#### Auditory Stimuli

We manipulated *stimulus construction* and *signal features* following a 2 × 2 design (Fig. 1b). In simple conditions, signals were pure tones. In the consistent condition, the signal was always a 440 Hz tone. In the alternating condition, the signal was either a 440 or 660 Hz tone. Tones were presented at 45 dB SPL. In complex conditions, signals were noise sounds. To generate a new sound on each trial, Gaussian noise (i.e., a sequence of normally-distributed random numbers) was filtered with a 2^nd^ order Butterworth bandpass filter. In the consistent condition, we always used edge frequencies of 1.0/1.1 kHz. In the alternating condition, we used edge frequencies of either 1.0/1.1 kHz or 1.1/1.2 kHz. Noise sounds were presented at 45 dB SPL. Noise sounds were presented within background noise (1^st^ order Butterworth bandpass filter, edge frequencies: 0.5/2.4 kHz, 50 dB SPL). Auditory signals had a ramp onset of 10 ms.

#### Visual Stimuli

Signals covered the area of a notional annulus with an inner/outer radius of 1°/4° (visual angle) around central fixation. In simple conditions, signals were composed of 3 concentric rings (1° increments, alternating black-to-white; Fig. 1b). In the consistent condition, the ring pattern always started on black. In the alternating condition, the ring pattern started either on black or white. In complex conditions, 1000 dots (1 pixel; black or white) were uniformly-distributed within the annulus as visual background stimulation. Each dot moved linearly in a random direction and speed (mean: 1°/s, *SD:* 0.2°/s). Dots were randomly replaced within the annulus when moving outside the annulus, or when the lifetime of 0.1 s had passed. For the signal, 50% of the dots started coherent rotation (mean: 0.67 rad/s, *SD*: 0.067 rad/s). In the consistent condition, the direction was always clockwise. In the alternating condition, the direction was either clockwise or anticlockwise. All visual stimuli were presented on a grey screen.

### Procedures

Each trial started with a central, green fixation point (0.27°). In complex conditions, both the auditory and visual background were presented as well. The foreperiod duration consisted of a fixed component (1 s) and an exponentially-distributed random component (mean: 0.75 s). Then, a signal was presented for a max. duration of 1.5 s. On catch trials, no signal was presented and stimulation as during the foreperiod continued for 1.5 s. After a response, or at the end of the max. duration, the fixation point changed from green to red to indicate that the trial ended. Following false alarms and misses, a feedback screen indicated the mistake for 2 s. Stimulation, including the background in complex conditions, stopped for 0.25 s before the next trial started.

Stimuli were presented in blocks of 104 trials (26 trials per modality, 26 catch trials). The trial sequence was randomised, but always started with an additional dummy trial (presenting redundant signals). After a false alarm or miss, a corresponding trial was repeated, the trial sequence was reshuffled, and stimulation continued with another dummy trial. Dummy trials were not analysed. The experiment included 16 blocks (2 × 2 within-subjects design with 4 blocks per condition). Conditions were intermixed in the randomisation of block order, which was done for each participant using a Latin square. A block lasted approx. 4 min (a small pointer on fixation denoted progress). The entire experiment lasted around 105 min with breaks.

### Data analysis

Any response within 1.5 s after signal onset was recorded as valid. Responses were false alarms when given during the foreperiod or at any time during catch trials. Signal trials with no response were misses. Before the main analysis, we performed an outlier correction. For this, we transformed the RTs of each condition into rates (1/RT). We excluded data points that deviated by more than 3 × 1.4826 × median absolute deviations (MADs) from the median^28^, corresponding to 3 SDs if data are normally distributed. Fast outliers occurred on 0.4% (±0.1) of trials and slow outliers on 0.5% (±0.1) of trials. A total of 24,720 valid RTs (approx. 103 per condition and participant) remained.

The main analysis focused on the speed-up of RTs with redundant signals compared to the unisensory components, which is the RSE. As defined previously, this multisensory benefit is best measured by the area between the cumulative distribution function (CDF) in the multisensory condition and the faster of the unisensory CDFs^12^. As a simple procedure, if the number of measured RTs is the same in all three conditions, the size of the area can be estimated:

1 $$benefit=\frac{{\sum }^{}min({A}_{i},{V}_{i})-A{V}_{i}}{N}$$

*A*_*i*_, *V*_*i*_, and *AV*_*i*_ are quantiles of the empirical CDFs, where the index *i* indicates the rank ranging from 1 (the fastest) to *N* (the slowest RT).

This simple computation requires equal numbers of RTs in each condition to allow for equal quantile points. However, following outlier correction, the numbers of RTs are often unequal. As a solution, we used linear interpolation^29^ to down-sample the collected RTs to a common sample size (we used 50 quantile points; the procedure was shown to be unbiased using Monte Carlo simulations). We computed empirical benefits for each participant and each condition of the 2 × 2 design.

To obtain quantitative predictions of the RSE, we used Raab’s model^18^. Based on the CDFs in the unisensory conditions, a parameter-free prediction of the CDF in the multisensory condition can be obtained using probability summation:

2 $${P}_{AV}(t)={P}_{A}(t)+{P}_{V}(t)-{P}_{A}(t)\times {P}_{V}(t)$$

*P*_*A*_ and *P*_*V*_ are the empirical CDFs in the unisensory conditions (we used linear interpolation to obtain continuous CDFs). *P*_*AV*_ is here the predicted CDF for multisensory condition. We then extracted 50 quantile RTs from the predicted CDF and calculated expected benefits analogous to the calculation of empirical benefits (Equation (1)).

Next, we quantified two processing interactions that go beyond Raab’s model. First, RTs on a given trial can depend on previously presented signals. To quantify this history effect, we computed the RT difference between trials in which modalities were repeated, and trials in which modalities were switched:

3 $$history\,effect={\overline{RT}}_{Switch}-{\overline{RT}}_{Repetition}$$

We computed the history effect for auditory and visual RTs separately and averaged these values to obtain a single measure. Second, we computed violations of Miller’s bound^8^, which is given by the sum of the CDFs in the unisensory conditions:

4 $${P}_{Miller}(t)=min[{P}_{A}(t)+{P}_{V}(t),1]$$

The CDF in the redundant signals condition has to fall below Miller’s bound if both the race model and context invariance hold^19^. To quantify violations, we first used linear interpolation to obtain 50 quantiles for Miller’s bound (*Miller*_*i*_). Then, similar to a previously detailed method^30^, we computed the size of the area between the CDF in the redundant condition and Miller’s bound, where the redundant condition exceeded the bound:

5 $$violation=\frac{\sum max(Mille{r}_{i}-A{V}_{i},0)}{N}$$

*AV*_*i*_ are the 50 quantile RTs from the empirical CDF in the redundant condition as used in the computation of benefits.

Finally, we applied the context variant race model^12,13^. All modelling was performed in the rate space (1/RT). As a first step, we fitted a LATER model^31,32^ to each of the unisensory conditions. As the LATER model assumes that rates are normally distributed, we used the Matlab function *normfit* to obtain best-fitting values of the mean (*Mu)* and the standard deviation (*Sigma)* using the Minimum-Variance Unbiased Estimator (MVUE). As a second step, we fitted the context variant race model in the redundant condition. Using a race mechanism, the model assumes that a response on a given trial with redundant signals is triggered by the unisensory signal with the higher rate. Hence, the resulting rate distribution can be computed using the maximum distribution of two Gaussian random numbers^33^, which are given by the LATER fits in the unisensory conditions. The exact distribution depends on the correlation (*Rho*), which we kept as a free parameter to account for history effects. As a second free parameter, the additional noise (*Eta*) increases the variability of rates (i.e. the *Sigma* parameter) in both LATER units additively. This free parameter manifests a violation of the context invariance assumption, which allows for violations of Miller’s bound. We used the Matlab function *mle* to obtain maximum likelihood estimates (MLEs) of *Rho* and *Eta* individually for each participant and each condition of the 2 × 2 design.

Statistical analyses were performed in IBM SPSS Statistics 22. For main analyses, we used 2 × 2 repeated-measures ANOVAs, which always tested the factors stimulus construction (simple, complex) and signal features (consistent, alternating). For initial analysis of median RTs, we used 2 × 2 × 3 repeated-measures ANOVAs, including the additional factor signal modality (A, V, and AV). We used a Greenhouse-Geisser correction where sphericity was violated. The alpha level for statistical testing was 0.05.

## Results

### General Performance

To assess data quality, we first checked performance (false alarms, misses). In signal trials, the false alarm rate during the foreperiod was 1.04% (±0.19%, SEM). In catch trials, the false alarm rate was 1.53% (±0.25%). The miss rate was 0.46% (±0.19%). As performance was close to perfect, it is not further considered in the RT analysis (for a detailed analysis of false alarm and miss rates, see Supplementary Analysis 1).

To assess whether the experimental manipulations mimicked the diversity of RSE studies, we next tested median RTs **(**Table S1**)**. A 2 × 2 × 3 repeated-measures ANOVA showed a significant main effect of stimulus construction, *F*(1, 19) = 154.59, *p* < 0.001, *ηp*^2^ = 0.89. RTs were faster with simple (0.300 ± 0.010 s) than with complex stimuli (0.416 ± 0.015 s). Hence, the experimental factor stimulus construction successfully manipulated RTs. There was neither a main effect of signal features nor any interaction (all *F* ≤ 2.04, p ≥ 0.165, *ηp*^2^ ≤ 0.10). Expectedly, there was a main effect of signal modality, *F*(1.39, 26.37) = 53.32, *p* < 0.001, *ηp*^2^ = 0.74. Pairwise comparisons revealed that redundant RTs (0.321 ± 0.011 s) were faster than both auditory (0.371 ± 0.014 s) and visual RTs (0.382 ± 0.012 s), both *p* < 0.001. Auditory and visual RTs were not significantly different, *p* = 0.626. This multisensory benefit is the RSE, which we investigate in full detail using our comparative approach.

Additionally, we also examined effects on variability of RTs, as measured by the Median Absolute Deviation (MAD) of RTs. A 2 × 2 × 3 repeated-measures ANOVA showed a main effect of stimulus construction, *F*(1, 19) = 58.851, *p* < 0.001, *ηp*^2^ = 0.76. MAD of RTs was smaller with simple (0.040 ± 0.003 s) compared to complex (0.060 ± 0.004 s) stimuli. There was also a main effect of signal modality, *F*(1, 19) = 35.306, *p* < 0.001, *ηp*^2^ = 0.65. As expected from probability summation, redundant RTs were less variable (0.040 ± 0.003 s) than auditory (0.057 ± 0.004 s) and visual (0.054 ± 0.004 s) RTs, both *p* < 0.001. MAD of auditory and visual RTs was not significantly different, *p* = 0.822. Finally, there was a significant interaction between stimulus construction and signal modality, *F*(2, 38) = 5.230, *p* < 0.010, *ηp*^2^ = 0.22. Paired-samples were conducted for MAD (pooled across signal features) for each modality. This analysis found larger MAD values for complex conditions compared to simple conditions for all three modalities, all *p* < 0.001. The interaction is explained by the difference between simple and complex MAD values being larger for auditory (0.021 s) and visual (0.024 s) signals than it is for redundant signals (0.013 s).

### Step 1: Multisensory Benefits

**Figure 2:**
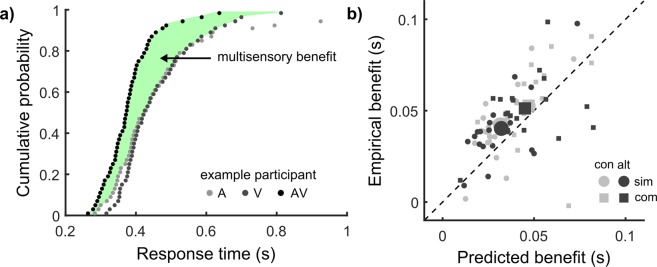
Analysing multisensory benefits. (**a)** Empirical benefits are measured by the area between the cumulative RT distributions in the multisensory (AV) condition and the faster of the unisensory conditions (A, V; Equation (1)). Data from an example participant in the complex-alternating condition. (**b)** Empirical benefits as a function of benefits predicted by Raab’s model (Equation (2)). Each point represents a participant in one of the four conditions. Large symbols represent the group mean.

As first step, we tested the ability of the race model framework to predict multisensory benefits, which are best measured based on RT distributions (Equation (1); for an illustration, see Fig. 2a). According to Raab’s model, a parameter-free prediction of the RT distribution with redundant signals can be obtained using probability summation (Equation (2)), which allows calculation of predicted benefits analogous to empirical benefits. For predicted benefits (Supplementary Fig. S1), a 2 × 2 repeated-measures ANOVA showed a main effect of stimulus construction, *F*(1, 19) = 41.54, *p* < 0.001, *ηp*^2^ = 0.69. Predicted benefits were larger for complex (0.046 ± 0.004 s) compared to simple stimuli (0.032 ± 0.003 s). No other effects were significant (all *F* ≤ 0.80, *p* ≥ 0.383, *ηp*^2^ ≤ 0.04). Hence, this simple analysis based on the unisensory RTs predicts larger benefits for complex compared to simple stimuli (which is here basically driven by the variability rule, see Supplementary Analysis 2).

We tested next if the RSE followed the prediction. For empirical benefits (Supplementary Fig. S1), a 2 × 2 repeated-measures ANOVA showed a main effect of stimulus construction, *F*(1, 19) = 5.87, *p* < 0.026, *ηp*^2^ = 0.24. Empirical benefits were larger for complex (0.052 ± 0.003 s) compared to simple stimuli (0.041 ± 0.004 s). No other effects were significant (all *F* ≤ 0.41, *p* ≥ 0.528, *ηp*^2^ ≤ 0.02). Hence, empirical benefits were consistent with the prediction.

To assess the explanatory power, we correlated predicted and empirical benefits (Fig. 2b). All correlation coefficients were positive, and significant in 3 of 4 conditions (Table S2). One notable observation is that mean empirical benefits for each participant over all conditions (0.047 ± 0.003 s) was larger than mean predicted benefits (0.039 ± 0.003 s). This was confirmed by a paired-samples *t*-test, *t*(19) = 3.07, *p* = 0.006. Thus, while Raab’s parameter-free model broadly predicts the RSE, it does not provide a complete account.

### Step 2: Empirical Interactions

As second step, we focus on two processing interactions that are not covered by Raab’s model. Firstly, we measured history effects by analysing RTs as a function of previously presented signals (Equation (3)). Expectedly, a 2 × 2 repeated-measures ANOVA showed a significant intercept of 0.034 s (±0.004 s), *F*(1, 19) = 75.62, *p* < 0.001, *ηp*^2^ = 0.80 (Fig. S3). Hence, unisensory RTs following a modality switch were slower compared to a repetition. If history effects arise simply due to the repetition of low-level signal features (e.g. the specific frequency of a tone), introducing alternating features within each modality should reduce history effects compared to consistent features. However, no effects were significant (all *F* ≤ 4.13, *p* ≥ 0.056, *ηp*^2^ ≤ 0.179). Hence, while clearly present and thus to be considered by any model of the RSE, the history effect did not change across conditions here (for additional tests, see Supplementary Analysis 3).

**Figure 3:**
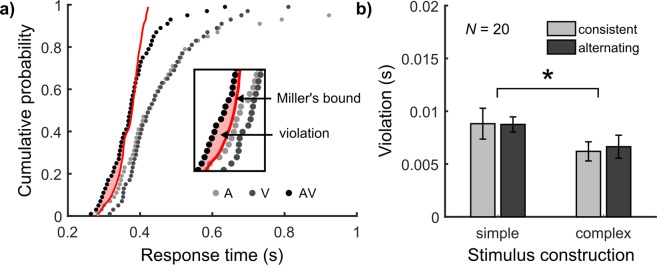
Measuring empirical interactions. (**a)** Violations of Miller’s bound are measured by the area between the cumulative RT distributions in the multisensory (AV) and the sum of the unisensory conditions (Equations (4) and (5)). Example data as in Fig. 2a. (**b**) Violations of Miller’s bound across conditions. Mean and SEM of 20 participants.

Secondly, we measured violations of Miller’s bound (Equations (4) and (5); for an illustration, see Fig. 3a). A 2 × 2 repeated-measures ANOVA showed a significant intercept of 0.008 s (±0.001 s), *F*(1, 19) = 130.70, *p* < 0.001, *ηp*^2^ = 0.87 (Fig. 3b). Hence, in agreement with most RSE studies using audio-visual signals, Miller’s bound was violated. In addition, there was a significant effect of stimulus construction *F*(1, 19) = 4.56, *p* < 0.046, *ηp*^2^ = 0.19. The violation area for simple stimuli (0.009 ± 0.001 s) was larger than for complex stimuli (0.006 ± 0.001 s). No other effects were significant (all *F* ≤ 0.05, *p* ≥ 0.819, *ηp*^2^ < 0.01). Thus, violations are clearly present and therefore must be considered by race models.

### Step 3: Modelling Approach

**Figure 4:**
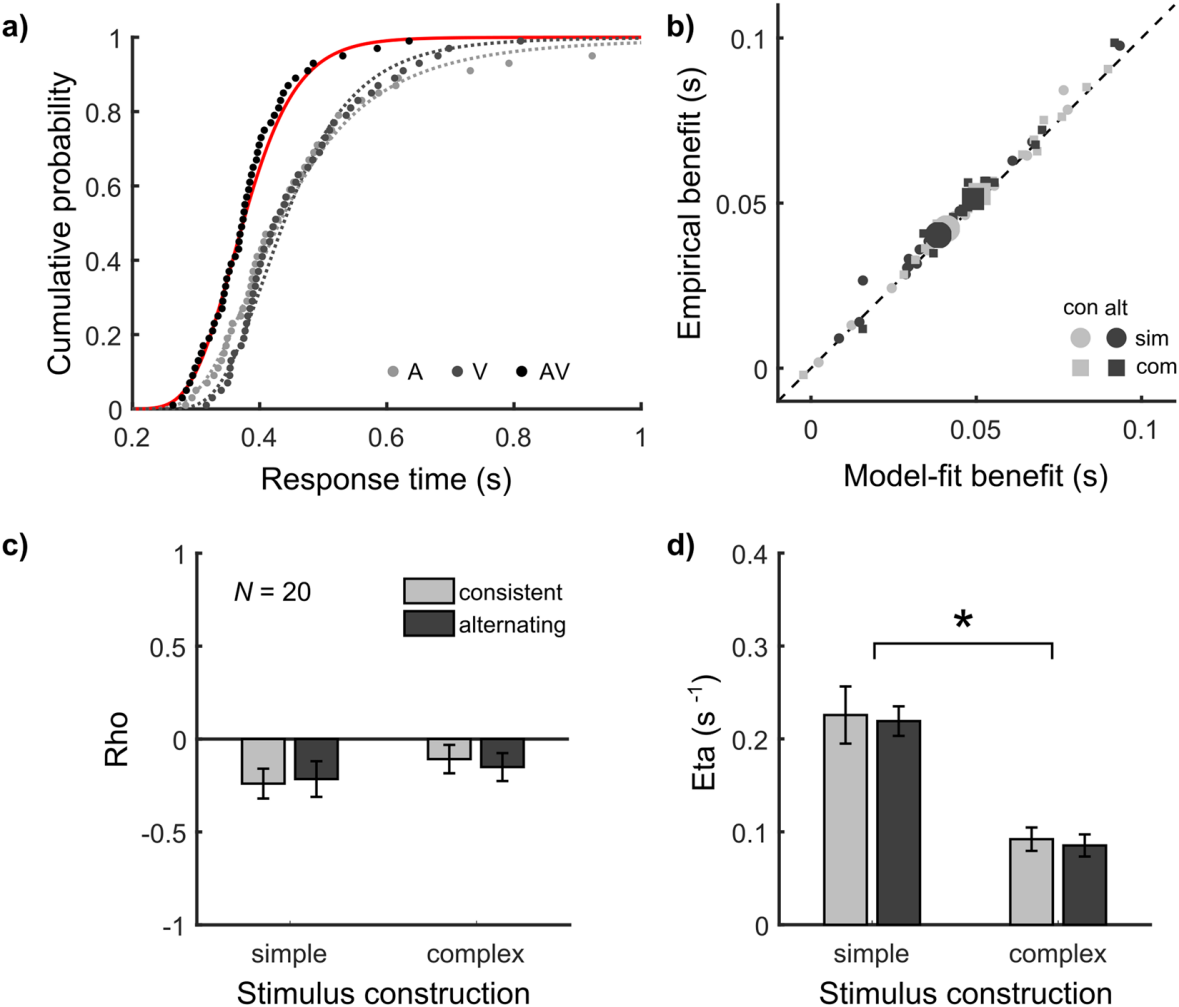
Model fitting. (**a)** Best-fitting context variant race model (solid line). The model is constrained by the fits of the LATER model in the unisensory conditions (dashed lines) and has the correlation *Rho* and the additional noise *Eta* as free parameters. Example data as in Fig. 2a. (b) Empirical benefits as a function of benefits calculated from the best-fitting context variant race model. Each point represents a participant in one of the four conditions. Large symbols represent the group mean. (**c**,**d)** Best fitting values of *Rho* and *Eta* across conditions. Mean and SEM of 20 participants.

As final step, we followed a modelling approach that accounts for the empirical interactions. We used the context variant race model^12,13^, which includes the correlation *Rho* and the noise *Eta* as free parameters to account for history effects and violations of Miller’s bound, respectively. We fitted the model on the level of RT distributions (for an illustration, see Fig. 4a). To assess if the best-fitting model fully explained the RSE, we computed benefits according to the model-fit analogous to empirical benefits (Equation (1)). As with the parameter-free predictions, we correlated model-fit and empirical benefits (Fig. 4b). The model explained the empirical benefits almost perfectly as all correlation coefficients were positive and highly significant (see Supplementary Table S2). Hence, the context variant race model provides a viable explanation for the RSE across conditions.

We next inspected the best-fitting model parameters with respect to the empirical interactions. Regarding the first parameter *Rho*, a 2 × 2 repeated-measures ANOVA showed a significant intercept of −0.178 (±0.064), *F*(1, 19) = 7.72, *p* = 0.012, *ηp*^2^ = 0.29 (Fig. 4c). Hence, there was overall a negative correlation between unisensory RTs. There were no further significant effects (all *F* ≤ 1.24, *p* ≥ 0.279, *ηp*^2^ ≤ 0.06). This finding is in agreement with the above finding that history effects did not differ across conditions. Regarding the second parameter *Eta*, a 2 × 2 repeated-measures ANOVA showed a significant intercept of 0.156 s^−1^ (±0.012 s^−1^), *F*(1, 19) = 176.06, *p* < 0.001, *ηp*^2^ = 0.90 (Fig. 4d). The parameter manifests a violation of the context invariance assumption, which allows for violations of Miller’s bound. We neither found a main effect of signal features nor an interaction (all *F* ≤ 0.15, *p* ≥ 0.708, *ηp*^2^ ≤ 0.01). Interestingly, there was a main effect of stimulus construction, *F*(1, 19) = 44.62, *p* < 0.001, *ηp*^2^ = 0.70. *Eta* was larger with simple (0.222 ± 0.019 s^−1^) compared to complex stimuli (0.089 ± 0.010 s^−1^). In agreement with the above finding that the violation of Miller’s bound is larger for simple compared to complex stimuli, more noise was required to account for the redundant RT distributions with simple compared to complex stimuli according to the model. The analysis of *Eta* thus points to a striking dissociation between the processing interactions revealed here (Fig. 4d) and multisensory benefits, which in contrast were larger with complex compared to simple signals (Supplementary Fig. S1).

## Discussion

To mimic the diversity of RSE studies, we used a factorial design to create distinct audio-visual stimulus sets. With our sets, RTs were faster for simple compared to complex stimuli (with a rather substantial RT difference of 0.116 s). The comparative approach outlined in this paper, therefore, can be considered a blueprint to systematically analyse the RSE with distinct stimulus sets or different participant groups, which we think is critically needed to gain complete understanding of the RSE.

As a first step to this approach, we focused on multisensory benefits as given by the size of the RSE. Initially, we made a quantitative prediction of the RSE based on Raab’s model^18^. At this point, we would like to highlight that this basic race model is fully constrained by the empirical RT distributions in the unisensory conditions, and does not contain free parameters. The quantitative prediction is simply computed based on probability summation (Equation (2)). Despite its simplicity, the model correctly predicted the increased RSE as observed for complex rather than simple stimuli (Fig. 2) and accounts considerably for the variation across participants (Table S2). To the best of our knowledge, no such straightforward predictions are provided by any published pooling model, which makes it difficult to understand why this alternative model class is favoured in RSE research e.g.^34^. In contrast, we argue that race models are a very promising candidate for a common framework for the diversity of RSE studies. At least, Raab’s model provides a simple tool for analysing the RSE when distinct signal sets or different participant groups yield different RTs.

As second and third step, we analysed two empirical interactions not accounted for by Raab’s model, and applied consequently the context variant race model, which is an extended race model including two free parameters to cover the interactions^12,13^. The first interaction concerns history effects, which challenges statistical independence (as assumed in Equation (2)). In agreement with previous studies^4,8,13^, we found significant history effects across conditions, which thus must be accounted for by any model of the RSE. In the race model, we dropped the statistical independence assumption and included instead the correlation *Rho* as a free parameter. As history effects are opposite for vision and audition (e.g. after presentation of a visual signal on the previous trial, fast visual and slow auditory RTs are expected), the best-fitting *Rho* value covered the effect by assuming a negative correlation (Fig. 4c).

One simple hypothesis, which we began with, is that history effects may arise due to the repetition of low-level signal features. For example, history effects may arise in auditory trials because tones with identical frequencies repeatedly stimulate the same population of sensory neurons. If this repeated stimulation is causally-linked, the history effect should be reduced when tones with different frequencies stimulate different populations of sensory neurons. Following this simple explanation, we expected to observe a main effect of signal features, whereby alternating signals would reduce history effects compared to consistent signals. However, such changes occurred neither for the measured history effect nor for the corresponding model parameter *Rho*. This suggests rather a basis in higher-level processes. For instance, in the field of task-switching^35^, costs are observed by switching between two tasks in the same modality. History effects may be the result of similar mechanisms (i.e. switching between auditory and visual detection tasks). Alternatively, in the attention literature, RT costs have been observed between targets in different modalities^36^. History effects may also arise from attentional mechanisms, whereby the previous unisensory trial directs attention to one modality at the expense of the other. Future investigations should develop manipulations to target such higher-level processes.

The second interaction concerns violations of Miller’s Bound, which challenges the context invariance assumption^19^. In agreement with most audio-visual RSE studies e.g.^4,5,8,9,13^, we found significant violations of Miller’s bound across conditions (Fig. 3b), which thus must be accounted for by race models. In the context variant race model^12,13^, we dropped the context invariance assumption and included instead the additional noise *Eta* as a second free parameter. Critically, for violations of Miller’s bound we found a main effect of stimulus construction, with increased violations occurring with simple compared to complex stimuli. The best-fitting *Eta* value covered the effect by assuming further increased noise in the evidence accumulation process for simple compared to complex conditions (Fig. 4d).

Previously, we have only speculated about potential sources of this additional noise^19^. Here, however, one aspect of the experimental factor of stimulus construction is that simple stimuli are expected to trigger strong onset transients, which are masked by the background stimulation in complex conditions. As onset transients have been considered relevant for multisensory processing^22,23^, one possibility is that the additional noise interaction is linked to an interference caused by these transients. Although our behavioural data does not allow us to identify exact sources, one interpretation could be that strong neuronal activity linked to sudden signal onsets leaks as noise across sensory modalities and otherwise separate parallel processes.

As a more general outcome of our comparative approach, the systematic analysis of the RSE calls for a clarification of the rather vague term “multisensory integration”. On the one hand, the term has been defined operationally as “as a multisensory response (neural or behavioural) that is significantly different from the responses evoked by the modality-specific component stimuli”^37^. Following this definition, we found that the multisensory benefit, and hence integration, was larger with complex compared to simple signals. On the other hand, in research on multisensory RTs, multisensory integration is often said to occur only if Miller’s bound is violated (which is even considered “a psychometric benchmark of integrative processing”^38^). Regarding this definition, we found the opposite. Violations of Miller’s bound (and consistently the best-fitting *Eta* value) were larger with simple compared to complex signals. Hence, the two variables clearly dissociate and should not be described using the same term or share the same concept.

The vagueness of definitions may have contributed to the lack of a common framework for the RSE across studies. In contrast, our comparative approach, which is based on Raab’s original race model^18^, provides conceptual clarity. The basic model architecture, which consists of parallel decision units coupled by a logic OR operator, is convincing as it perfectly matches the task demands as imposed by the redundant signals paradigm^19^. Moreover, it provides a strong, parameter-free predictor of multisensory benefits^12^. As such, the combination rule for redundantly defined signals (or the multisensory integration mechanism, under an alternative conception) is clearly defined. Beyond this basic mechanism, at least two very specific processing interactions emerge and can be precisely quantified by explicit parameters in a model-based approach.

The approach is general enough to be applied to various experimental setups and research questions. This includes not only experiments comparing different sensory modalities, such as audio-tactile^4^ or visual-tactile stimulation^5^, but also studies that investigate unisensory RSE^39,40^ or processing differences between uni- and multisensory versions of the redundant signals paradigms^41^. The approach is also suitable to shed light on processing differences between participant groups. For instance, studies on ageing typically show larger multisensory benefits for older compared to younger participants^9^. Such findings may be directly understood if older participants’ RT distributions are more variable, as unisensory RT variability is the driving force behind multisensory benefits according to race models^12^. Finally, since the framework has successfully been used to study dyslexia^42^, it could of course also be applied to study other clinical populations, such as those with Schizophrenia^43^ or Parkinson’s Disease^44^. In the aggregate, we believe that our comparative approach provides both a general account and the precision needed to target and understand processing differences across diverse studies, which are key features for a common explicatory framework of the RSE.

Beyond the RSE, we believe this work contributes to a broader effort to achieve clarity of definitions in multisensory research^37^. In the wider literature, there are many instances of behavioural paradigms and associated measures assumed to reflect ‘multisensory integration’ equivalently. As shown here within one paradigm, however, this assumption can be incorrect. We hope therefore to demonstrate for multisensory researchers the importance of defining measures in detail and verifying their proposed relationships experimentally. Failing to do so may result in similar confusion when comparing results between paradigms.

## Supplementary information

Supplementary Information Document

## Data Availability

The research data supporting this publication can be accessed at 10.17630/c8cbd7b7-e2b3-4e62-bb3d-66fce081ff59^45^. The data analysis and model-fitting tools are also freely available online via the RSE-box^46^: https://github.com/tomotto/RSE-box.
