# Supplementary material for: A comparative analysis of response times shows that multisensory benefits and interactions are not equivalent
Source: Sci Rep. 2019 Feb 27;9:2921. doi: 10.1038/s41598-019-39924-6 (PMC6393672; doi:10.1038/s41598-019-39924-6)
Supplement: Supplementary file 1 — Supplementary Information Document [file 41598_2019_39924_MOESM1_ESM.pdf]

Supplementary Information

A comparative analysis of response times shows that multisensory benefits and interactions are not equivalent

Bobby R. Innes & Thomas U. Otto
School of Psychology and Neuroscience, University of St. Andrews

Supplementary Inventory:

Supplementary Analysis 1 (False alarm and miss rates)
Supplementary Analysis 2 (Principles of multisensory behaviour)
Supplementary Analysis 3 (History analysis)

Figure S1 (Multisensory benefits)
Figure S2 (Principles of multisensory behaviour)
Figure S3 (History analysis)

Table S1 (Median RTs)
Table S2 (Model vs. empirical benefits)

Supplementary Analysis 1

False Alarms

In signal trials, the false alarm rate during the foreperiod was 1.04% (±0.19%, SEM). A 2×2×3 ANOVA (stimulus construction × signal features × signal modality) showed no effects (all $F \leq 4.11$, $p \geq .057$, $\eta p^2 \leq .18$). In catch trials, the false alarm rate was 1.53% (±0.25%). A 2×2 ANOVA (stimulus construction × signal features) showed a significant interaction, $F(1, 19)=4.77$, $p=.042$, $\eta p^2=0.20$. However, no significant differences were revealed in follow-up paired-samples *t*-tests (all $p \geq .118$). No further effects were significant (all $F \leq .02$, $p \geq .878$, $\eta p^2 < .01$).

Misses

The miss rate was 0.46% (±0.19%). A 2×2×3 ANOVA revealed a main effect of stimulus construction, $F(1, 19)=5.23$, $p=.034$, $\eta p^2=0.22$. Misses were more frequent for complex (0.78% ±0.32%) than for simple stimuli (0.14% ±0.08%). There was also a significant effect of signal modality, $F(1.211, 23.011)=4.03$, $p=.050$, $\eta p^2=.18$. Misses for redundant signals (0.14 ±0.08%) were smaller than for visual signals (0.50% ±0.18%, $p=.010$) but not auditory signals (0.73 ±0.33%, $p=.123$). Misses for auditory and visual signals did not differ ($p=.906$). No other effects were significant (all $F \leq 3.69$, $p \geq .061$, $\eta p^2 \leq .16$).

Supplementary Analysis 2

Directional predictions of the RSE for different sets of signals can be readily made according to the *principles of multisensory behaviour* [1]. Firstly, the *principle of equal effectiveness* states that benefits should increase when unisensory RTs become more similar. To assess the principle of equal effectiveness, we used the absolute difference between the median RTs to auditory and visual signals:

$$median\ difference = |\widetilde{RT}_A - \widetilde{RT}_V| \quad \text{(S1)}$$

Benefits are expected to decrease if the median difference increases. A 2×2 ANOVA on the median difference (**Fig. S2a**) showed no significant effects (all $F \leq 2.42$, $p \geq .136$, $\eta p^2 \leq .11$). Hence, according to the first rule, benefits should not change.

Secondly, the *variability rule* states that the variability of RTs is the key driving force of multisensory benefits, which should increase when unisensory RTs become more variable. To assess the variability rule, we used the MAD of RTs in the unisensory condition that was less variable:

$$minimum\ MAD = min(MAD(RT_A), MAD(RT_V)) \quad \text{(S2)}$$

The minimum MAD was chosen here as increasing variability in the RTs to a second unisensory signal does not lead to further benefits [1, their Fig. 3]. A 2×2 ANOVA showed a main effect of stimulus construction, $F(1, 19)=116.59$, $p<.001$, $\eta p^2=.86$ (**Fig. S2b**). The minimum MAD was larger with complex (0.060 ±0.004 s) than with simple stimuli (0.040 ±0.003 s). No other effects were significant (all $F \leq .41$, $p \geq .532$, $\eta p^2 \leq .02$). Hence, as variability is the driving force of multisensory benefits according to race models, benefits are expected to increase in complex compared to simple stimuli.

Discussion

Empirical benefits shown in the main paper are well in line with the directional forecast provided by the principles of multisensory behaviour [1], which predicted larger benefits with complex signals due to the variability rule (**Fig. S2**). Regarding the principle of equal effectiveness, no differences were

expected in our experiment. The principle was however put to test by another recent paper, which adapted participants to different delays between audio-visual signals [2]. This study found that largest multisensory benefits occurred for conditions in which the unisensory response onset asynchrony (i.e. the difference between unisensory RTs) was smallest, which we would say perfectly follows the forecast by the principle of equal effectiveness. Together, these results add support for the principles of multisensory behaviour as a simple diagnostic tool for understanding the RSE.

References

1 Otto, T. U., Dassy, B. & Mamassian, P. Principles of Multisensory Behavior. *J Neurosci* **33**, 7463-7474, doi:10.1523/Jneurosci.4678-12.2013 (2013).

2 Harrar, V., Harris, L. R. & Spence, C. Multisensory integration is independent of perceived simultaneity. *Exp Brain Res* **235**, 763-775, doi:10.1007/s00221-016-4822-2 (2017).

Supplementary Analysis 3

Given that the variability of RTs is the driving force of the RSE according to race models, it is critical to understand how much the history effect (**Equation (3)**, **Fig. S3a**) contributes to the overall variability of unisensory RTs. To assess this contribution, we computed the *history index*, which normalises the history effect with the variance of RTs:

$$history\ index = \frac{history\ effect^2}{var(RT)} \tag{S3}$$

As with the history effect, we computed the history index separately for auditory and visual RTs, and averaged then to obtain a single measure (**Fig. S3b**). A 2×2 ANOVA, however, showed that no effects were significant (all $F \leq 2.91$, $p \geq .104$, $\eta p^2 \leq .13$).

Discussion

The lack of a main effect of signal features here shows that the history effect did not contribute differently to the overall variability in consistent compared to alternating conditions. This experimental factor consequently manipulated neither the history effect nor the history index. Hence, these effects seem not to depend on the repetition of low-level stimulus features (such as the frequency of a tone).

Supplementary Figures

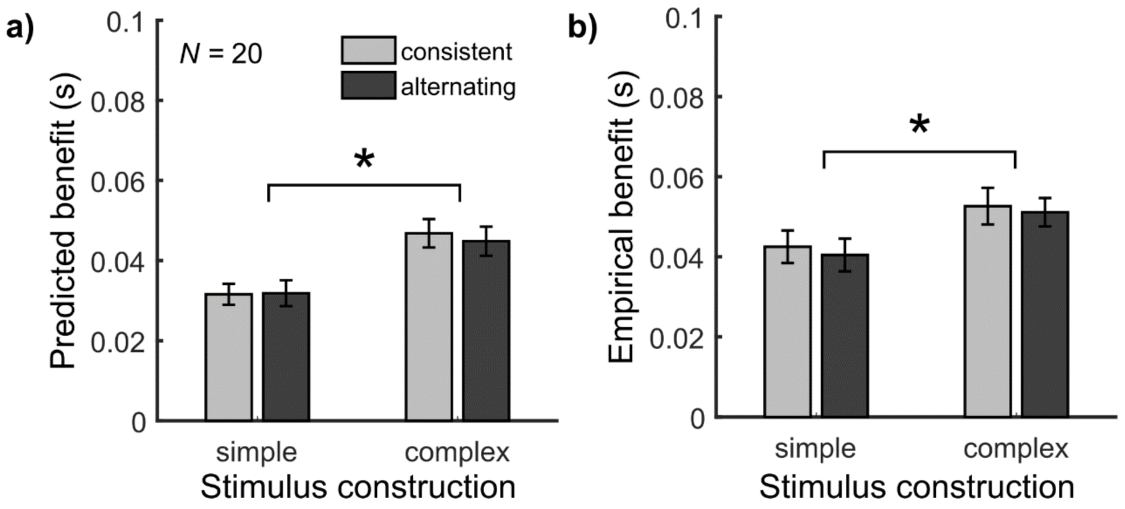

Figure S1. Multisensory benefits. **a)** Benefits predicted by Raab's model and **b)** benefits calculated based on empirical RT distributions, as a function of stimulus construction and signal features. Mean and SEM of 20 participants.

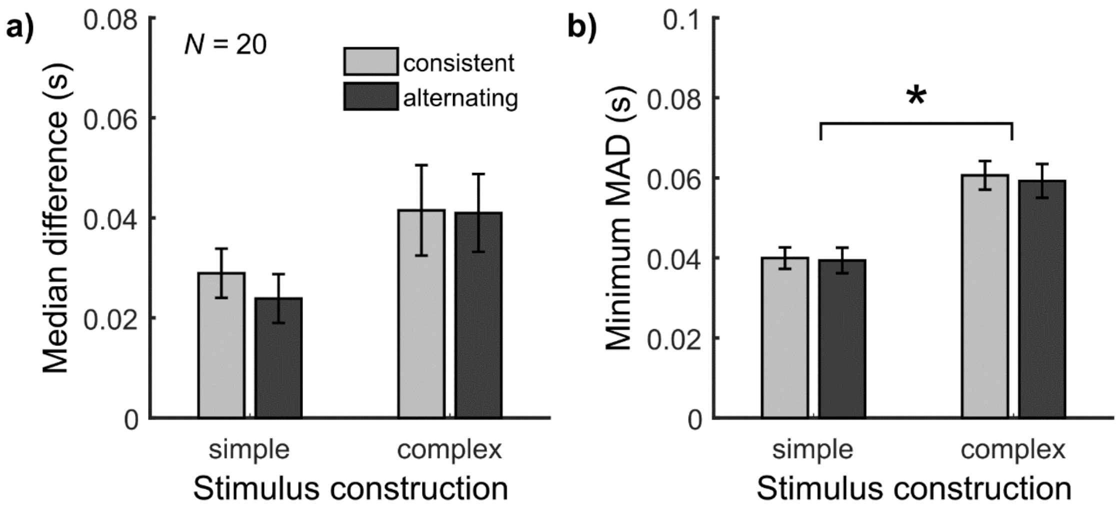

Figure S2. Principles of multisensory behaviour. **a)** Median difference (**Equation (S1)**) and **b)** Minimum MAD (**Equation S2**) within unisensory conditions, as a function of stimulus construction and signal features. Mean and SEM of 20 participants.

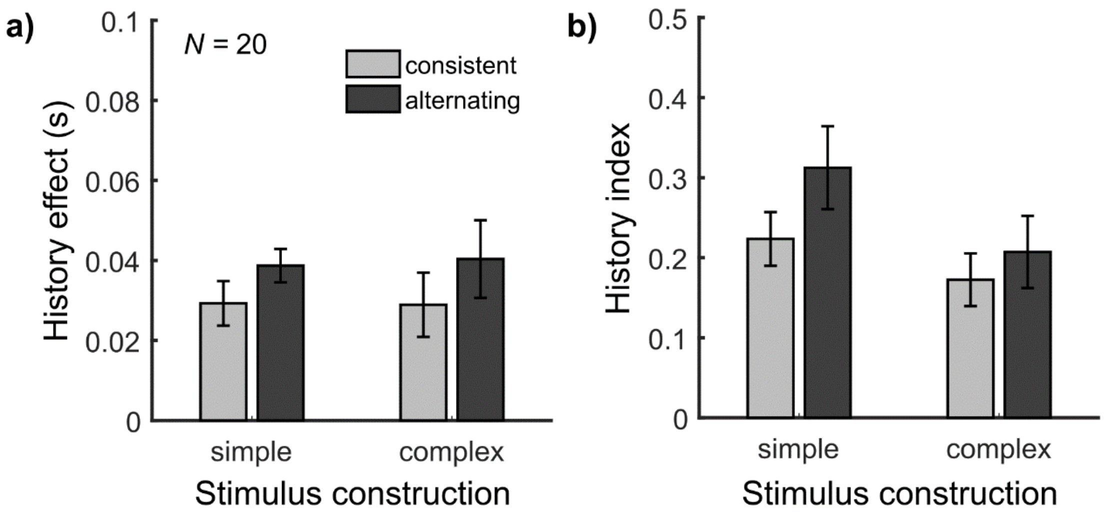

Figure S3. History analysis. **a)** History effect (**Equation (3)**) and **b)** history index (**Equation (S3)**) as a function of stimulus construction and signal features. Mean and SEM of 20 participants.

Supplementary Tables

Table S1. Median RTs (s). The table shows the mean (SEM) of 20 participants.

stimulus construction	signal features					
	consistent			alternating		
	**A**	**V**	**AV**	**A**	**V**	**AV**
simple	0.311 (0.013)	0.320 (0.008)	0.265 (0.007)	0.314 (0.014)	0.320 (0.009)	0.272 (0.010)
complex	0.427 (0.018)	0.444 (0.019)	0.372 (0.016)	0.433 (0.017)	0.443 (0.016)	0.375 (0.014)

A auditory, V visual, AV redundant (see Figure 1)

Table S2. Model vs. empirical benefits. The table shows Pearson correlation coefficients (and p-values).

stimulus construction	predicted vs empirical (Figure 2b)		model-fit vs empirical (Figure 4b)	
	signal features		signal features	
	**consistent**	**alternating**	**consistent**	**alternating**
simple	0.731 (p<.001)	0.690 (p=.001)	0.995 (p<.001)	0.992 (p<.001)
complex	0.447 (p=.048)	0.332 (p=.152)	0.996 (p<.001)	0.986 (p<.001)
